# The effect of essential oil of *Zataria multiflora* incorporated chitosan (free form and Pickering emulsion) on microbial, chemical and sensory characteristics in salmon (*Salmo trutta*)

**DOI:** 10.1016/j.fochx.2023.100999

**Published:** 2023-11-22

**Authors:** Nooshin Zomorodian, Shahrzad Javanshir, Nabi Shariatifar, Sadegh Rostamnia

**Affiliations:** a Department of Chemistry, Iran University of Science and Technology, Tehran, Iran; b Department of Environmental Health Engineering, School of Public Health, Tehran University of Medical Sciences, Tehran, Iran

**Keywords:** Antimicrobial properties, Antioxidant activity, Fish, Microbiological analysis, Seafood

## Abstract

The objective of current research was to prepare a new biodegradable coating containing chitosan (Ch) and *zataria multiflora* essential oil (ZMEO) (free and Pickering emulsion (PEO) forms), in order to enhance the *Salmo trutta* shelf life. Our results showed, the mean of films thickness, mechanical properties (elastic modulus (EM) and tensile strength (TS) analysis) and WVP in different treatments were ranged from 0.103 ± 0.003 (for Ch) to 0.109 ± 0.003 (for Ch-PEO (2.5 %)) µm for thickness, from 3.2 ± 1.6 (for Ch) to 8.15 ± 2.3 (for Ch-EO) MPa for EM, from 1.3 ± 0.5 (for Ch-EO) to 1.6 ± 0.06 (for Ch) Mpa for TS and from 0.1 ± 0.02 (for Ch) to 0.8 ± 0.05 (for Ch-EO) (×10 − 11(g m/m2 s Pa) for WVP. In current research, the lowest and highest total viable counts (TVC) was related to Ch-PEO (1.7 log CFU/g) and control treatments (4.65 log CFU/g). The lowest and highest of pH was related to the Ch-PEO (6.45) and the control (7.1), the lowest and highest of PV (peroxide value) was related to Ch-PEO (0.34 meq/kg) and control treatment (1.37 meq/kg), the lowest and highest of TBARS (thiobarbituric acid reactive substances) was related to Ch-PEO (0.37 mg/kg) and control treatment (2.23 mg/kg) and also the lowest and highest of TVB-N (total volatile base nitrogen) was related to Ch-PEO (17.7 mg) and control (59 mg). Also, Ch-PEO showed the best sensory properties after sixteen days. Among all the treatments in all the tests, the best maintenance property was related to the Ch-PEO, therefore, chitosan coatings containing ZM Pickering emulsion should be considered as a potential active coating in the fish industry.

## Highlights

• Evaluation the chitosan coating with *Zataria multiflora* EO to protect Trout fish.
• The coating were prepared in two forms of free EO and Pickering emulsion.
• Physicochemical, microbial and sensory changes of treatments were evaluated.
• The treatment of Ch-PEO had the highest maintenance effect.

## 1 Introduction

The incidence of foodborne diseases has been increasing globally, even in developed countries with high health standards. However, many cases of food poisoning go unreported, making it difficult to accurately track the amount of illness caused by consuming rotten meat, especially in developing countries (Abdollahzadeh et al., 2014, Keykhosravy et al., 2020; Nabi Shariatifar, Fathabad, & Madihi, 2016). Seafood, particularly fish, is prone to spoilage due to transportation and improper storage. Fish are highly susceptible to microbial spoilage and fat oxidation due to their poor oxidative stability and special compounds like unsaturated fatty acids and amino acids (De Groot, 1991, Jung et al., 2009). *Salmo trutta,* a type of fish native to western Asia, Europe, and north-western Africa, is commonly found in freshwater and coastal marine environments, primarily in rivers and lakes. (Ferguson et al., 2019, Klemetsen et al., 2003).

There are various methods to increase the shelf life of food, including freezing, canning, heating, drying, irradiation, and salting. However, researchers are now exploring new techniques like active packaging using edible coatings and biodegradable films, along with the use of antimicrobial compounds and natural antioxidants. (Cui et al., 2022, Mokarami et al., 2022).

Edible coatings are thin layers of edible materials that act as a natural barrier against moisture, oxygen, and aroma. These coatings can be applied to food through immersion, spraying, or brushing to create a modified atmosphere. It is important for these coatings to be made from food-safe and food-grade materials. The goal is to develop coatings that can extend the shelf life of food without compromising its quality. Films made from natural additives like essential oils and extracts can act as active coatings, carrying antimicrobial substances and antioxidants. Plants essential oils (EO) and extract (E) (among natural additives) are of interest in the field of green consumerism due to their antioxidant and antimicrobial properties (Raeisi et al., 2014, Sayadi et al., 2021; Y. Shahbazi and Shavisi, 2018, Sharifimehr et al., 2019).

*Zataria multiflora Boiss (Lamiaceae)*, a plant found in Pakistan, Afghanistan, and Iran, is known for its antioxidant and antimicrobial properties. In Iran, it is called ‘‘Avishane Shirazi’’ and used as a flavoring agent in food and has been studied for its various health benefits, including analgesic, immune-stimulating, anti-inflammatory, antiviral, antifungal, antibacterial, and antioxidant effects. These films offer a green alternative for consumers interested in sustainable products. (Keykhosravy et al., 2020, Keykhosravy et al., 2021, Rahimabadi et al., 2012).

Chitosan (Ch) is a polysaccharide derived from chitin, which is found in the exoskeleton of invertebrates and is abundant in nature. It is commonly obtained from crustaceans like crabs and shrimp, as well as certain fungi and algae. Chitosan can form a film and is used as a coating for food, as well as for other natural polymers. It has unique properties such as the ability to absorb heavy metal ions and antimicrobial effects. These properties can help reduce food oxidation, increase food safety, and extend the shelf life of food (Mokarami, Arabameri, Shariatifar, & Shiran, 2022).

Emulsions are mixtures where small droplets of one liquid are dispersed in another liquid. Biphasic dispersion emulsions use an emulsifier to reduce surface tension and stabilize the mixture. Pickering emulsion is a nanoemulsion technique that uses solid particles to increase stability (Keykhosravy et al., 2021, Shen et al., 2021, Sun et al., 2020). Nanotechnology, a small-scale engineering field, has revolutionized agriculture and food industries. By controlling nanoscale particle size, properties like texture, taste, sensory qualities, and stability can be altered in food products. (Pabast et al., 2018, Shen et al., 2021).

Consumers of prepared foods prioritize the safety, health, and quality of their food. To address these concerns, producers can utilize natural antibacterial and antioxidant substances derived from plants. These substances not only help maintain the quality of fish but also extend its shelf life. By incorporating these plant-based ingredients, producers can create suitable and high-quality products, thereby addressing consumer concerns effectively (Hasani et al., 2020, López-Caballero et al., 2005). Also, in the past, a number of researchers showed the effect of different treatments on *Salmo trutta* fish, among which we can refer to the research of Yumuk et al. (that analyzed effects of chitosan with vegetable oil on shelf life of brown trout (*Salmo trutta fario*) fillets) (Yumuk, Alak, & Yanik, 2019) and Zarabi et al. (that analyzed the preservation effect of ice-glazing using pullulan and bay laurel extract on the quality characteristics of caspian Trout (*Salmo trutta caspius*) during frozen storage) (Zarabi, Ahmadi, Hedayatifard, Golestan, & Farhadi, 2022) in this field. Considering the high consumption of fish among humans, and on the other hand, since so far, a comprehensive and detailed study (in Iran and the world) in the field of Trout fish preservation using edible coating containing chitosan and *Zataria multiflora* essential oil (in Pickering emulsion and free form) has not been done, the present study seems very necessary. Therefore, the main objective of this research was to investigate a novel coating containing chitosan and essential oil of *Zataria multiflora* in free form and Pickering emulsion to protect Trout (*Salmo trutta*) against the chemical (pH, PV, TBARS and TVB-N), microbial (TVC), physical (TS (tensile strength) and EM (elastic modulus), water vapor permeability (WVP) and thickness), and also sensory (red color, discoloration and off-odor) changes during storage at 4 °C for 16 days.

## 2 Materials and methods

### 2.1 Materials

From Merck Co. (Darmstadt, Germany), acetic acid, zein, ethanol, folin-Ciocalteu reagent, glycerol (>97 % purity), sodium carbonate anhydrous, Ch powder with a deacetylation degree of 75–85 percent (medium weight of molecular (MW: 190e310 kDa)) were obtained. Other solvents and reagents were of analytical grade or higher available purity.

### 2.2 Plant material and EO preparation

The *Zataria multiflora* leaf was obtained from the market of Shiraz, Iran, and at Tehran University of Medical Sciences (TUMS) by a pharmacognosy specialist, was authenticated. According to the technique mentioned by the Pabast et al., *Zataria multiflora* leaf was washed (with distill water) and in the shade air- dried at temperature of ambient and hydro distillated for three hours, using an apparatus of all-glass Clevenger-type (Pabast, Shariatifar, Beikzadeh, & Jahed, 2018). Finally, the prepared essential oil, over anhydrous sodium sulphate was dried and kept at 4 °C in sealed vials until being applied.

### 2.3 The identify essential oil components by GC/MS

Agilent Technologies model 7890A was GC equipment and Detector of Mass Selective was 5975C VL with Triple-Axis Detector. The GC–MS conditions and specifications were data processed by Agilent MSD Chemstation (Rev E.02.02.1413); Analyzer: Quadrupole; Column: Rtx five MS (I.D.: 0.250 mm, Length: 30 m and Film thickness: 25 μm); ion Source: Electron Impact (EI) 0 eV; Carrier Gas: He 99.999 %; Temperature of Ion Source: 230 °C; Injection Port Temperature: 230 °C; Program Rate (°C/min): 3; Sample Volume: 0.2 μL; Temperature of Initial (°C): 40; Final Temperature (°C): 270; Initial Time: one minute; Final Time: ten minutes; Septum purge (ml/min); Split ratio: 100 mL/min; and Flow rate (ml/min): 1.

### 2.4 Preparation of Pickering emulsion *Zataria multiflora* essential oil

For this purpose, zein (1 g) was added to ethanol (70 mL) and stirred, and afterward, deionized water (130 mL) drop by drop was added, and then it was placed in a bath of ultrasonic to completely dissolve, and then was placed in a rotary device at a 45 °C temperature and mixed at 50 rpm (to thicken) for two hours. Then the final volume of the solution reached 100 mL and was divided into two parts of 50 mL each. Fifty microliters of ZMEO was added to one of the samples and 250 Âµl was added to the other sample, then both samples were homogenized with a mixer at a speed of 12000 rpm (Almasi et al., 2020, Shen et al., 2021, Sun et al., 2020).

### 2.5 Preparation of chitosan solution

One percent chitosan (w/v) was dissolved in 1 % acetic acid (w/w) and mixed at 325 rpm at 50 °C for 1 h (Homayounpour et al., 2020, Pabast et al., 2018).

### 2.6 Preparation of edible film

One percent chitosan (w/v) was dissolved in one percent acetic acid (w/w) along with distilled water and glycerol as a plasticizer and mixed at 350 rpm for 3 h at a temperature of 40 °C on a magnetic hotplate mixer. In the next step, 40 mL of the obtained solution was poured into two Petri dishes with a ten centimeters diameter, and then ZMEO and ZM-PEO were added to the mixture in a 5 % (w/w) mixture and left for 48 h. It was dried at a temperature of 25 °C and retained at a temperature of 25 °C (in a desiccator) until the experiments (Homayounpour et al., 2020, Pabast et al., 2018).

### 2.7 Thickness and mechanical properties

All methods were according to the Qin et al. study (Qin, Li, Liu, Yuan, & Li, 2017). By using a digital micrometer (Mitotuyo 7327, Tokyo, Japan), the film’s thickness was measured. From 10 replications across each film sample was calculated the mean value. For mechanical properties, by using a CMT 4104 tensile testing equipment (MTS Systems Co., Ltd, Shanghai, China) were assessed TS (tensile strength) and EM (elastic modulus) of the films.

### 2.8 Water vapor permeability (WVP)

The films WVP was analyzed based on the standard method of ASTM E 96–95 by using a measuring cup of water vapor transmission filled with the desiccant as it was explained in the other study. Five replications were tested for each film sample, and was calculated the mean value (Qin, Li, Liu, Yuan, & Li, 2017).

### 2.9 Scanning electron microscopy (SEM)

According to the study of Behbahani et al. were assessed the structure and morphology of film, by using SEM analyzer ((KYKY-EM3200; KYKYTechnology Development Ltd.,Beijing, China) (Behbahani, Noshad, & Falah, 2019).

### 2.10 Fourier transform infrared spectroscopy (FTIR) analyses

Spectral measurement parameters of resolution and accumulation assessed by FTIR analysis. It can assess characteristics of chemical and provide detailed information of compositional. The FTIR of film was assessed according to the study of Cebi et al. (Cebi, Arici, & Sagdic, 2021).

### 2.11 Samples preparation

Fish tissue was obtained from the market 48 h, posts slaughter. Next step, by using sterile cutting boards and knives, 96 steaks (about weight of 60 g) (thick of 2 cm and dimensions of 6 × 6 cm) were cut (aseptically). Until being coated by the technique of immersion, each fish steak samples were placed into a tray (polystyrene) and preserved in the temperature of the refrigerator (4 °C). Next to the preparing of solutions, fish steak samples were divided (randomly) into 4 groups (i) control or uncoated (Con); (ii) coated containing with solution of solely chitosan (Ch); (iii) coated containing with Ch and ZMEO solution (Ch-EO); (iv) coated containing with Ch and ZM-PEO and each treatment was immersed for one minute in the solutions of corresponding. The mentioned treatments were dipped for another one minute in the solutions of coating, in order to achieve a uniform coating layer on the surface of fish samples. After that, the fish samples were permitted to drip additional solution. For fifteen minutes, all treatments at 25 °C were dried. The treatments were placed into plastic sterile Petri dishes (individually) and were hermetically sealed and retained for up to 16 days in the refrigerator at 4 °C. From each four treatments, 3 samples were investigated on six days (1, 4, 7, 10, 13 and 16) (Homayounpour et al., 2020, Pabast et al., 2018).

### 2.12 Microbiological analysis

By using plate count agar (PCA, Merck code 1.05463, Darmstadt, Germany), the TVC (total viable counts) were assessed, after incubation at 30 °C for three days (Homayonpour et al., 2021, Pabast et al., 2018).

### 2.13 Determination of pH

This method was carried out by homogenizing approximately 10 g of samples with twice the amount of distilled water (g/ml). A pH meter was used to determine the pH value (model HI 2002, HANNA Company, Germany). Experiments were performed at least three times (Homayonpour et al., 2021, Pabast et al., 2018).

### 2.14 Thiobarbituric acid reactive substances (TBARS)

The TBARS index was assessed based on reaction between thiobarbituric acid (TBA) with secondary lipid oxidation products like ketones and aldehydes. In this study, the TBARS value was evaluated according to the previous studies, and reported as mg of malonaldehyde (MDA) equivalents/kg of treatments (Homayonpour et al., 2021, Mehdizadeh et al., 2021, Pabast et al., 2018).

### 2.15 TVB‑N (total volatile base nitrogen value)

The TVB-N is related to food spoilage specially seafood. The TVB-N of the fish was assessed by using the technique of the micro diffusion as defined by Raeisi et al. and reported as mg N/100 g of treatment (Raeisi, Hashemi, Aminzare, Ghorbani Bidkorpeh, Ebrahimi, Jannat, et al., 2020). The fish samples (10 ± 0.1 g) were homogenized in 100 mL of perchloric acid (6 %) for two minutes (in a suitable container) and then filtrated. Then it was alkalized with sodium hydroxide solution (20 %) and finally steam distillation of the extract is done. By an acid receiver, the volatile base components were absorbed and determined by titration. All analyses were carried out in triplicate. This parameter is generated (generally) by enzymatic and bacterial degradation of fish proteins and non-protein compounds of nitrogen (N_2_). The TVB-N value is a generic term that contains ammonia, trimethylamine, dimethylamine, and other compounds of volatile N_2_.

### 2.16 Peroxide value (PV)

The value of peroxide showed the peroxides formed in the primary stage of oils and fats oxidation. The value of PV was calculated and represented as meq O_2_/kg fat (Homayounpour et al., 2020, Pabast et al., 2018).

### 2.17 Sensory evaluation

For sensory evaluation, according to the previous method, treatments were assessed by 9 semi-trained panelists (Homayounpour et al., 2020, Pabast et al., 2018), which all of them had an experience in fish evaluation and earlier contributed in another research conducted by (Homayounpour et al., 2020, Pabast et al., 2018) at Tehran University of Medical Sciences. The analyzed sensory parameters were: ‘discoloration’, ‘red color’ and ‘off-odor’, which used a 5-point descriptive scale by using a questionnaire according to the previous mentioned method (Homayounpour et al., 2020, Pabast et al., 2018). The number one indicated the highest score (best quality) and the number five indicated the lowest score (lowest quality).

### 2.18 Statistical analysis

The statistical analyses of the data were done by two-way ANOVA (P < 0.05) using the general linear model procedure to determine the main effects time, different treatments (control, Ch, Ch-EO and Ch-PEO), and their interaction (time × treatments) on microbial, physical, chemical and microbial parameters. The Statistical Package for the Social Sciences (SPSS) (Chicago, IL)). All examinations were run in triplicate, data are exhibited as means ± SD (standard deviations).

## 3 Result and discussion

### 3.1 Analysis of essential oil compounds

After extraction of essential oils by hydrodistillation, GC/MS was used to identify the volatile compounds in it. The volatile compounds is listed in Tables 1, respectively. Twenty-two compounds of essential oil make up 98.82 % of the identified compounds. According to this table, the main recognized components were Thymol (37.5 %) is one of the main compounds of thyme essential oil. Both thymol and thyme essential oil have long been used in traditional medicine as expectorant, anti-inflammatory, antiviral, antibacterial, and antiseptic agents, Gamma-Terpinene (19.2 %) has antimicrobial properties against various pathogens, Para-Cymene (12.2 %), Trans-Caryophyllene (10.2 %) and Carvacrole (6.3 %). In 2022, Dadazadeh et al. analyzed essential oil compounds of *Zataria multiflora* and reported the most recognized components (in order of prevalence) were carvacrol, thymol, linalool, p-cymene, decane, β-caryophyllene and γ-terpinene, which were analogous (somewhat) with our results (Dadazadeh & Nourafcan, 2022).

**Table 1:** Analysis of essential oil compounds by gas chromatograph / mass spectrometry (GC/MS).

Peak NO	Compounds	RT(min)	A%
1	Alpha-Tujene	7,2	1.07
2	Alpha-Pinene	9	1.6
3	Beta-Pinene	9,23	0.3
4	Camphene	11	1.9
5	Alpha.Phellandrene	11.24	0.3
7	3-Carene	11.5	0.1
8	Beta-Pheilandrene	11.6	0.6
9	Beta-Myrcene	11.8	1.63
10	Linalool	12	0.8
11	Para-Cymene	12,7	12.2
12	Alpha-Terpinolene	13	0.71
13	Carvacrole	14,4	6.3
14	1,8Cineole	15	0.3
15	Gamma-Terpinene	15.8	2.6
16	Alpha-Terpinolene	16.13	0.1
17	Thymol	23	37.5
18	Trans-Caryophyllene	30.5	10.2
19	Alpha-Humulene	32.4	0.6
20	Spathulenol	37	0.11
21	Caryophyllene Oxide	37.5	0.7
22	Gamma-Terpinene	49	19.2

and mostly occurs in thyme species.

### 3.2 Thickness, mechanical (EM and TS) properties and WVP

An efficacious parameter for analyze water barrier and mechanical properties of films is thickness. According to the Table 2, our result showed that the mean of films thickness in different treatments was ranged from 0.103 ± 0.003 (for Ch) to 0.109 ± 0.003 (for Ch-PEO (2.5 %)) µm. The thickness of the layers listed in Table 2 has increased significantly (p < 0.05) due to the addition of ZMEO and its percentage in the film solution, which may be due to the intramolecular interaction between chitosan polymeric chains and free essential oil compounds, increasing the density of the film and a factor for increasing the thickness of films containing free essential oil. By another study, the increase of film thickness owing to the adding agent of antioxidant active has also been stated (Peng, Wu, & Li, 2013). Almasi et al. assessed the active packaging film made from pectin activated by nano-emulsion and Pickering-emulsion stabilized EO of marjoram (*Origanum majorana L*.) and reported that the thickness of control and other treatments was ranged from 99.66 to 158.43 µm, which was higher than our results (Almasi, Azizi, & Amjadi, 2020).

**Table 2:** Thickness, mechanical properties and WVP (water vapor permeability) of film (mean ± SD).

Treatments	WVP (×10^−11^(g m/m^2^ s Pa)	EM (elastic modulus) MPa	TS (tensile strength) MPa	Thickness (µm)
Ch (chitosan)	0.1 ^A^ ± 0.02	3.2 ^A^ ± 1.6	1.6 ^A^ ± 0.06	0.103 ^A^ ± 0.003
Ch-EO (chitosan-essential oil)	0.8^B^ ± 0.05	8.15 ^D^ ± 2.3	1.3^C^ ± 0.5	0.104 ^A^ ± 0.003
Ch-PE (chitosan- Pickering emulsion) (0.5 %)	0.6^C^ ± 0.06	5.1^C^ ± 2.3	1.33^C^ ± 0.5	0.108^B^ ± 0.003^B^
Ch-PE (2.5 %)	0.48 ^D^ ± 0.07	4.03^B^ ± 2.1	1.4^B^ ± 0.5	0.109^B^ ± 0.003

*Different letters in the column represent statistically significant differences among the four treatments (P < 0.05).

Our result also showed that (Table 2) the mean of EM and TS analysis as mechanical properties, were ranged from 3.2 ± 1.6 (for Ch) to 8.15 ± 2.3 (for Ch-EO) MPa and from 1.3 ± 0.5 (for Ch-EO) to 1.6 ± 0.06 (for Ch) Mpa, respectively. Pure chitosan film sample has higher tensile strength than other treatments. Pure chitosan film has better tensile strength than films containing ZMEO and ZM -PEO. The film’s TS decreased after adding ZMEO to the matrix of polymer, the addition of ZMEO reduced significantly the elasticity (P < 0.05). Wu et al. in 2015, stated this interaction leads to a weak mechanical response (Wu, Liu, Ge, Wang, Qin, Chen, et al., 2015). In general, the treatments containing ZM-PEO had better TS and better elongation % than the treatments comprising ZMEO. Also, by adding ZMEO to the film and also by enhancing its level, the value of TS significantly reduced (P < 0.05) that can be interpreted as the compounds in the herbal essential oil by being in between the polymer chains, the bonds within the polymer network are weakened, the structural density is disturbed and the matrix is destroyed. A similar behavior has been reported for the effect of adding Pickering emulsion to other composites (Huang and Yu, 2006, Roy and Rhim, 2021, Shen et al., 2021). Qin et al. analyzed the film of active packaging prepared from poly (lactic acid) incorporated EO (bergamot, lemongrass, rosemary, or clove), and reported that the TS and EM were ranged from 17 to 45.8 MPa and 623.9 to 1702.8 MPa, respectively that were higher than our findings (Qin, Li, Liu, Yuan, & Li, 2017).

The WVP displays the assessment of the films water barrier characteristics and their application in food packaging. The transfer of water vapor from films is related to the two factors of permeability of water molecules and solubility in the film structure. One of the principal factors of films (biodegradable) is the WVP. This parameter was set in order to examine the combined influence of ZMEO and ZM-PEO on the film of chitosan barrier characteristics. In this study, Table 2 exhibited that the permeability to water vapor in treatments containing EO and PEO was lower (significantly) than Ch treatment (0.1 ± 0.02 (×10^−11^ (g m/m2 s Pa)) (P < 0.05). Also, with increasing levels of ZM-PEO, the permeability to water vapor decreases (P < 0.05). But, with increasing levels of 2.5 % Pickering emulsions, the permeability to water vapor increased. In general, the lowest penetration rate was observed in Ch-EO treatment (0.8 ± 0.05 (×10^−11^ (g m/m2 s Pa)). The reduction of WVP in the treatments could be due to the presence of PEO, which is uniformly distributed in the polymer matrix. The WVP is one of the important practical characteristics of films which depends on the morphology of films, the type of plasticizer, the structure of chemicals, the conditions of measurement (like gradient of water vapor pressure and temperature) and the nature of permeability (Kristo et al., 2008, Niro et al., 2021). Ghanbarzadeh et al. stated that WVP was affected by the hydrophobicity- and hydrophobic properties of the film constituents (Ghanbarzadeh, MOUSAVI, REZAEI, Razmi, Milani, & OUROUMIEHEI, 2006). Qin et al. analyzed the film of active packaging prepared from poly (lactic acid) incorporated EO (bergamot, lemongrass, rosemary, or clove), and reported that the WVP value was varied from 1.06 to 2.03 (×10^−11^ (g m/m2 s Pa), which was higher than our results (Qin, Li, Liu, Yuan, & Li, 2017).

### 3.3 FTIR analysis

**Fig. 1:**
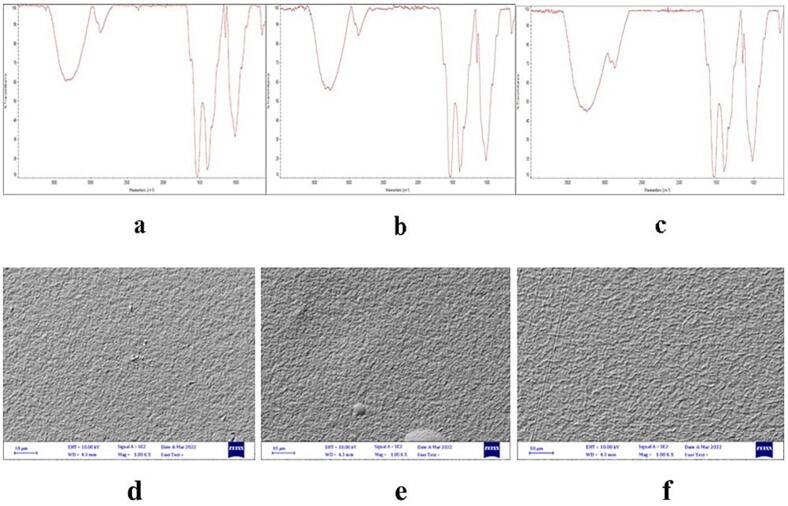
FTIR (fourier-transform infrared spectroscopy) analysis of treatments of chitosan (a), chitosan- Zataria multiflora EO (b) and chitosan- Zataria multiflora Pickering emulsion (c); and SEM (scanning electron microscopy) images of chitosan (d), Ch-EO (e) and Ch-PEO (f).

FTIR spectroscopy was used for the bioactive molecule composition of chitosan besides their distribution on the resulting chitosan. Results explain a wide-ranging absorption peak starts at 400–4000 cm^−1^ for chitosan (Fig. 1**a-c**). The results indicate the absorption peaks of chitosan and the interpretation of vibrational assignment with functional groups. The peaks at 1174 cm–1 is attributed to the C—O—C stretching, the peaks at 2865 cm–1 is attributed to the CH2 stretching, and the peaks at 3414 cm–1 is attributed to the OH stretching. The peaks at 1537 cm-1 and 1558 cm-1 are attributed to the CONH2 and NH2 groups, respectively. The FTIR spectroscopy was used for the bioactive molecule composition of chitosan- ZMEO besides their distribution on the resulting chitosan. Results explain a wide-ranging absorption peak starts at 400–4000 cm–1 for chitosan- ZMEO (Fig. 1). It is also observed the presence of absorption bands in the range of 2865 cm − 1 with a new band occurring in 2892 cm − 1, characteristics of the asymmetric and symmetrical vibrations of the CH2 group present in ZMEO, respectively. Absorption band displacement from 2865 cm − 1 (chitosan) to 2892 cm − 1 (chitosane/EO) was evidenced as a result of the interaction between the hydrophobic groups present in the chitosan/ ZMEO. FTIR spectroscopy was used for the bioactive molecule composition of ZM-PEO besides their distribution on the resulting chitosan. Results explain a wide-ranging absorption peak starts at 400–4000 cm^−1^ for ZMPEO (Fig. 1**a-c)**. As can be seen, the CH2 group is respectively present in the PE. Absorption band displacement from 2892 cm − 1 (chitosan/ZMEO) to 2912 cm − 1–2944 cm − 1 (chitosane/ZMPEO) was evidenced as a result of the interaction between the hydrophobic groups present in the chitosan/ ZM- PEO. Our results also confirmed by other studies (Ardekani et al., 2019, Bahrami et al., 2023, Nakhaee Moghadam et al., 2021, Torabiardekani et al., 2023).

### 3.4 SEM analysis

In this study, by using SEM electron microscope, the surface morphology of Ch, Ch-EO and Ch-PEO films was investigated. By using SEM, the nanoparticles dispersion level in the matrix, the composite homogeneity, the lumps presence, the void presence, and in some cases the orientation of nanoparticles was found. The SEM micrograph of the Ch film showed this was non-porous, void-free and smooth. Also, the results **(**Fig. 1**d-f)** showed that there was no large accumulation in the Ch-PEO and this shows the good dispersion of the Ch-PEO throughout the film matrix. This surface smoothness has also been observed to a large extent in the case of Ch-EO film. Similar outcomes have been reported in other researches on SEM micrographs (Li et al., 2022, Shahbazi et al., 2021, Sun et al., 2020).

### 3.5 Microbial analysis

#### 3.5.1 TVC test

**Fig. 2:**
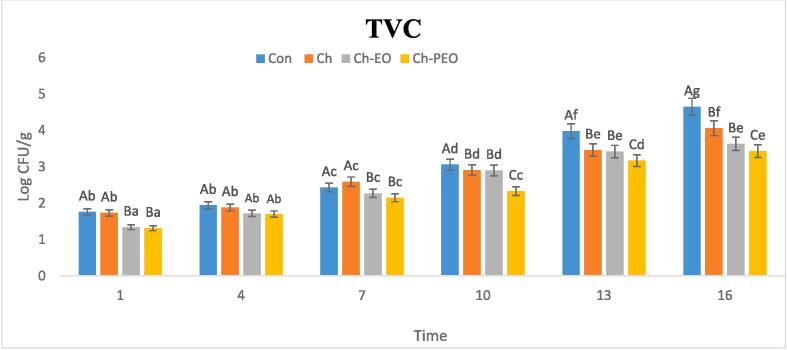
TVC (total viable counts) in different treatments.

Fig. 2, shows the changes of total viable counts (TVC) in different treatments during sixteen days. According to the figure, in different treatments, there was no difference in the total bacteria count on the first day of the research. On the first day, the amount of TVC was almost the same in all samples and the control group (between 1.31 and 1.76 log CFU/g). On the fourth day, the change in the TVC value in the other treatments and the control sample was different, so that the lowest total count of bacteria was related to chitosan treatment containing ZM-PEO (1.7 log CFU/g). The highest rate was related to control treatments (4.65 log CFU/g) and Chitosan (4.06 log CFU/g) on the 16th day. Also, on the 16th day, chitosan treatment had a TVC of 4.06 log CFU/g and Ch-EO treatment had a TVC of 3.63 log CFU/g. There is significant interaction (p > 0.05) between time and treatments of coated samples in the change of the TVC value (Table S1). The mechanism of chitosan to inhibit microbial growth leads to the polysaccharide polycationic nature that leads to the destruction of the cell membrane as well as the film formation around the bacterial cell. A change in the structure of the coating due to the rise of hydrophobic amino acid can form a coating with less hydrophilicity. Therefore, primary bacterial growth by ZMEO may promote antibacterial activity. Chitosan coatings containing Pickering emulsion showed stable and long-term antimicrobial activity compared to the coating containing ZMEO. Emulsifying phenolic compounds can increase the protect bioactive compounds from degradation, active agent’s availability, and their reaction with cells of microbial (Pabast et al., 2018, Peng et al., 2013; Y. Shahbazi and Shavisi, 2018, Wu et al., 2015). In similar study, Pabast et al. assessed effects of Ch coatings containing with free/nano form of *Satureja* EO on lamb meat quality characteristics and reported the TVC value was ranged between 4.85 log CFU/g (for CH-nano EO treatment) to upper than 7 for control samples (Pabast, Shariatifar, Beikzadeh, & Jahed, 2018). Also this similar results reported by other researchers (Chen et al., 2021, Homayounpour et al., 2020, Mahdavi et al., 2018).

### 3.6 Chemical analysis

#### 3.6.1 pH value

**Fig. 3:**
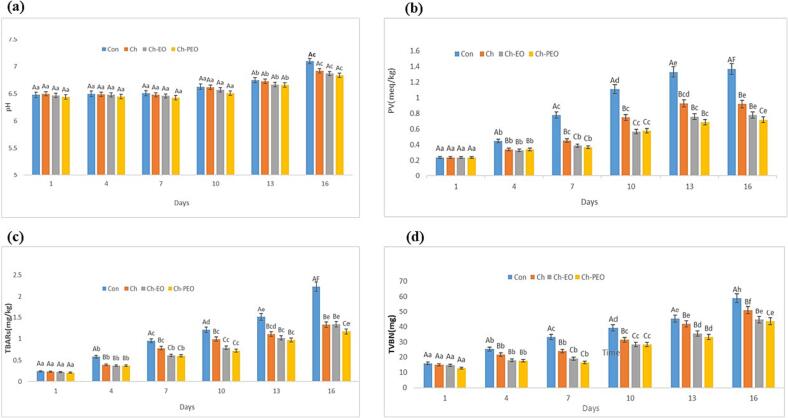
Changes in pH value (a), PV value (peroxide value) (b), TBARS (thiobarbituric acid reactive substances) value (c) and TVB-N (total volatile base nitrogen) value (d) during storage.

Fig. 3**a** shows on day one, there was no difference between the different treatments (between 6.44 and 6.5). On the fourth day, the pH change in the treatments and the control sample was different. The lowest pH was related to the chitosan containing ZM- PE on the fourth day (6.45) and the highest amount of pH was related to the control (7.1) and chitosan treatment (6.92) on the sixteenth day. Also, on the 16th day, the Ch-EO treatment had a pH of 6.87. There is difference (significant) in pH changes in the coated samples. There is significant interaction (p > 0.05) between time and treatments of coated samples in the pH changes (Table S1).The slight changes in the pH level of the coated samples may be due to the growth of lactic acid bacteria during the storage period and compensation of the increase in pH level through the amount of acid during growth. Also, the increase in the pH in fish samples might be associated with the autolysis process, production of alkaline matters (like indole, trimethylamine, ammonia, and histamine), endogenous enzymes (such as proteases and lipases) or microbial enzymes activity that leads to a rise in volatile bases during long-term storage. This research was analogous to the research of Homayonpor *et al.*, which they stated during storing fish at 4 °C, *Cuminum cyminum L*. nano-EO had better influence compared to the free-EO (Homayonpour, Jalali, Shariatifar, & Amanlou, 2021). EL-HANAFY *et al.* analyzed pH value on Nile tilapia (*Oreochromis niloticus*) fish and stated extract of green tea had better effect compared to the control fish sample (EL‐HANAFY, Shawky, & Ramadan, 2011). Also, Pabast *et al.* reported during storing fish at 4 °C, Satureja plant nano-EO had better influence on the pH of lamb meat compared to the free-EO, (Pabast, Shariatifar, Beikzadeh, & Jahed, 2018).

#### 3.6.2 PV assay

Fig. 3**b** displays the PV changes in four different treatments during sixteen days. According to the peroxide chart, there was no difference in different treatments on day one (0.24 meq/kg). On the fourth day, the change in the oxidation rate was different in the treatments and the control sample. As the results show, the lowest amount of oxidation was related to the treatment given with chitosan containing ZM Pickering emulsion (0.34 meq/kg) on fourth day and the highest amount was related to the control treatment (1.37 meq/kg) on the sixteenth day. Also, on the 16th day, the chitosan treatment had a PV of 0.92 meq/kg and the Ch-EO treatment had a PV of 0.78 meq/kg. There is significant interaction (p > 0.05) between time and treatments of coated samples in the pv changes (Table S1). The permanent changes in the amount of peroxide in the coated samples (free form and Pickering emulsion form of ZM) can be due to the phenolic and flavonoid substances release in the fish tissue during storage. Also, during the storing time, the peroxide value significantly raised in the fish samples, which was caused by the formation of fatty acids (short-chain) owing to the bacterial enzymes hydrolysis that is sensitive to oxidation possess and conducted to the formation of peroxides (Aşik & Candoğan, 2014; Homayounpour, Shariatifar, & Alizadeh‐Sani, 2021; Kizil et al., 2010, Pabast et al., 2018). Our results were similar to the other study (Homayounpour, Jalali, Shariatifar, Amanlou, & Khanjari, 2020; N Shariatifar et al., 2019, Tometri et al., 2020).

#### 3.6.3 TBARS assay

Fig. 3**c** displays the TBARS changes in four different treatments during sixteen days. According to the chart, thiobarbituric acid did not differ in different treatments on day one (0.21 to 0.24). On the fourth day, the change in the amount of thiobarbituric acid was different in different treatments. According to this figure, the lowest amount of thiobarbituric acid is associated to Ch-PEO (0.37 mg/kg) on fourth day and the highest amount is related to control treatment (2.23 mg/kg) on sixteenth day. Also, on the 16th day, chitosan treatment had TBARS 1.33 mg/kg and chitosan-essence treatment had TBARS 1.34 mg/kg. There is significant interaction (p > 0.05) between time and treatments of coated samples in the TBARS changes (Table S1).The reduction of thiobarbituric acid in chitosan treatment containing ZM- PEO is probably due to the controlled and slow release of essential oil on the fish surface. In fish samples, lipids peroxidation can be happening by photosensitized oxidation and autoxidation or by applying reaction enzymatic, like those relating to lipoxygenase and peroxidase, and bacterial enzymes (Aşik and Candoğan, 2014, Homayounpour et al., 2021, Kizil et al., 2010, Pabast et al., 2018). In a research by Zhang et al. in 2002, it was shown that the use of chitosan coating in pork meat stored at refrigerated temperature causes a reasonable reduction in fat oxidation and an increase in the quality and duration of meat storage (Zhang, Li, Kang, & Peng, 2022). Other studies, using chitosan coating and vegetable essential oil in beef, salmon, pork and lamb under conditions of cold storing, stated Ch coating samples comprising vegetable essential oil had lower TBA content than uncoated samples (Homayounpour et al., 2020, Pabast et al., 2018; Y. Shahbazi & Shavisi, 2018). Also, findings of current study was analogous to the study of Rahimabadi et al. (Rahimabadi, Rigi, Rahnama, Safari, Arshadi, & Barani, 2012) and Homayonpour et al. (Homayonpour, Jalali, Shariatifar, & Amanlou, 2021).

#### 3.6.4 TVB-N assay

Fig. 3**d** displays the changes of TVB-N in four different treatments during sixteen days. According to the chart, the changes of TVB-N on the first day were not different in different treatments (12.8 to 16 mg). On the fourth day, the change in the TVB-N value was observed in the coated samples and the control sample. The lowest TVB-N value was associated to Ch-PEO treatment (17.7 mg) on fourth day and the highest TVB-N value was associated to control (59 mg) and chitosan treatment (51 mg) on sixteenth day. And also on the 16th day, Ch-EO treatment had TVB-N 44.5 mg. The TVB-N in coated samples has significant (p > 0.05) difference between time and treatments (Table S1). Increasing in value of TVB-N is owing to the degradation of enzymes, proteins and derivatives in the seafood products, and a variety of volatile bases produced, such as H2S, histamine, trimethylamine and ammonia this product (Tometri, Ahmady, Ariaii, & Soltani, 2020). The findings of current research was analogous to the study of Tometri *et al.,* which they stated form *Laurus nobilis* leaf nano-extract had better effect compared to the free form (Tometri, Ahmady, Ariaii, & Soltani, 2020). In another study, Hasani *et al*. stated in TVB-N assay, nano-EO of lemon had better effect compared to the free form that this result confirmed our findings (Hasani, Ojagh, Ghorbani, & Hasani, 2020). EL-HANAFY *et al.* analyzed TVB-N value on Nile tilapia (*Oreochromis niloticus*) fish and stated treatment with green tea extract had better effect compared to the control fish sample, during storage (EL‐HANAFY, Shawky, & Ramadan, 2011). Also, Sayyari *et al.* analyzed TVB-N value with the free/nano EO of *Foeniculum vulgare* on fish fillets and confirmed our findings (Sayyari, Rabani, Farahmandfar, Esmaeilzadeh Kenari, & Mousavi Nadoshan, 2021).

### 3.7 Sensory evaluation

According to the Table 3, the samples were significantly affected by storage (p < 0.05). After seven days, the control sample achieved an unacceptable color score. The chitosan coating (Ch, Ch-EO and Ch-PEO) samples reached the highest acceptability red color score after 10 days. The chitosan sample containing ZMEO received a score of 3 after 13 days. In chitosan containing ZM -PEO, the fading of the red color was gradually delayed, even at the end of the time of storing, these fish samples did not reach grade 3. Therefore, after 16 days of storage in the refrigerator, Ch-PEO had an acceptable red color (p < 0.05). Control sample, chitosan-coated samples, chitosan-coated samples containing essential oil reached scores of 3 or higher, indicating unacceptable fading. On the day 10, the control sample and on days 10–13 the chitosan sample and on the 13th day the chitosan sample containing ZMEO did not reach the score of 3 at the end of the time of storing. The best conditions, protecting the fish sample from color change (discoloration), was the chitosan sample containing ZM-PEO. On the day 4 of storage, the control and chitosan treatment were given a foul odor score of 3, which corresponds to thiobarbituric acid values much higher than 2. The participants detect the oxidized odor, which is related to thiobarbituric acid in the range of 0.6–2, which delayed the odor formation until day 13 of the chitosan samples containing ZMEO. By giving a score of 1, the chitosan sample containing ZM-PEO was very effective in delaying the spread of bad odor and showed no discernible odor until the 16th day. Overall, Ch-EO and Ch-PEO increases the fish shelf life. But adding ZM-PEO to the coating was the most effective method to protect the fish red color, off-odor and discoloration, so that it was acceptable during storage until the last day of the fish (Table 3). According to previous research, it has been observed that the using of PEO technology raises the quality of sensory properties of products (Bazargani-Gilani, Aliakbarlu, & Tajik, 2015). Also, these outcomes were analogous to the study of Homayonpour *et al.* and Pabast *et al.* (Homayonpour et al., 2021, Pabast et al., 2018). Furthermore, EL-HANAFY *et al.* analyzed sensory properties of fish samples with green tea extract and reported compared to the control samples, this treatment could be retaining their good quality characteristics (EL‐HANAFY, Shawky, & Ramadan, 2011).

**Table 3:** Changes of Sensory in four different treatments during sixteen days.

Sensory parameters	Treatments	Days					
		1	4	7	10	13	16
**Red color**	**Control**	1	2	3	4	5	5
	**Ch (chitosan)**	1	1	1	1	4	5
	**Ch-EO (chitosan- essential oil)**	1	1	1	1	3	4
	**Ch-PEO (chitosan- Pickering emulsion)**	1	1	1	1	1	2
**Discoloration**	**Control**	1	2	3	4	5	5
	**Ch**	1	1	1	1	4	5
	**Ch-EO**	1	2	1	1	3	4
	**Ch-PEO**	1	1	1	1	1	2
**Off-odor**	**Control**	2	3	4	5	5	5
	**Ch**	1	3	3	4	4	5
	**Ch-EO**	1	1	1	2	3	4
	**Ch-PEO**	1	1	1	1	1	2

## 4 Conclusion

*Zataria multiflora* essential oil and ZM-PEO were added to the chitosan film. FT-IR and SEM results showed some new interactions between Ch and ZM. According to our findings, the WVP, and thickness of films were improved by adding ZMEO and ZM-PEO. However, the tensile strength of the composite films reduced by adding of ZMEO and ZM-PEO. Also, microbial and chemical evaluations showed that ZM-PEO in chitosan coating led to a delay in chemical and microbial spoilage and increased fish shelf life during storage at refrigerator temperature. Pickering emulsion of ZMEO allows the controlled release of bioactive agents on fish to increase antioxidant and antimicrobial activity during 16 days compared to free ZMEO, and these results indicate that chitosan coating with ZM-PEO can be applied as an active coating in the industry of seafood such as fish. Among the limitations of this research, we can point out the comparison of essential oil with water and ethanol extract, the use of different concentrations of essential oil in the research.

## CRediT authorship contribution statement

**Nooshin Zomorodian:** Writing – original draft, Methodology, Data curation. **Shahrzad Javanshir:** Conceptualization, Supervision, Validation, Writing – review & editing. **Nabi Shariatifar:** Conceptualization, Supervision, Validation, Writing – review & editing. **Sadegh Rostamnia:** Visualization, Investigation, Methodology, Software, Validation.

## Declaration of Competing Interest

The authors declare that they have no known competing financial interests or personal relationships that could have appeared to influence the work reported in this paper.

## Data Availability

No data was used for the research described in the article.
