# Supplementary material for: The effect of essential oil of Zataria multiflora incorporated chitosan (free form and Pickering emulsion) on microbial, chemical and sensory characteristics in salmon (Salmo trutta)
Source: Food Chem X. 2023 Nov 22;20:100999. doi: 10.1016/j.fochx.2023.100999 (PMC10740042; doi:10.1016/j.fochx.2023.100999)
Supplement: Supplementary data 1 [file mmc1.docx]

**Supplementary materials:**

**Table S1. Results of the two-factor (group by time) within ANOVA using the general linear model in different edible coating**

|  | Source | Type III Sum of Squares | df | Mean Square | F | Sig. |
| --- | --- | --- | --- | --- | --- | --- |
| TVC | treatments | 2.617 | 3 | .872 | 55272.382 | 0.000 |
|  | time | 35.615 | 5 | 7.123 | 451267.452 | 0.000 |
|  | treatments * time | 1.099 | 15 | .073 | 4639.741 | 0.000 |
| TVBN | time | 9977.871 | 5 | 1995.574 | 1906343.718 | 0.000 |
|  | treatments | 1315.579 | 3 | 438.526 | 418917.889 | 0.000 |
|  | time * treatments | 275.427 | 15 | 18.362 | 17540.772 | 0.000 |
| PV | treatments | 1.732 | 3 | .577 | 1167315.869 | 0.000 |
|  | time | 5.311 | 5 | 1.062 | 2147520.291 | 0.000 |
|  | treatments * time | .710 | 15 | .047 | 95642.157 | 0.000 |
| pH | treatments | .114 | 3 | .038 | 873.581 | 0.000 |
|  | time | 2.020 | 5 | .404 | 9248.681 | 0.000 |
|  | treatments * time | .072 | 15 | .005 | 109.978 | 0.000 |
| TBARS | treatments | 2.161 | 3 | .720 | 778892.953 | 0.000 |
|  | time | 13.452 | 5 | 2.690 | 2909158.436 | 0.000 |
|  | treatments * time | 1.238 | 15 | .083 | 89215.214 | 0.000 |
